# The Role of Proteolysis in Amyloidosis

**DOI:** 10.3390/ijms24010699

**Published:** 2022-12-31

**Authors:** Laura Acquasaliente, Vincenzo De Filippis

**Affiliations:** Laboratory of Protein Chemistry and Molecular Hematology, Department of Pharmaceutical and Pharmacological Sciences, University of Padova, 35131 Padova, Italy

**Keywords:** amyloidosis, proteolysis, protein aggregation, transthyretin, amyloid precursor protein, synuclein

## Abstract

Amyloidoses are a group of diseases associated with deposits of amyloid fibrils in different tissues. So far, 36 different types of amyloidosis are known, each due to the misfolding and accumulation of a specific protein. Amyloid deposits can be found in several organs, including the heart, brain, kidneys, and spleen, and can affect single or multiple organs. Generally, amyloid-forming proteins become prone to aggregate due to genetic mutations, acquired environmental factors, excessive concentration, or post-translational modifications. Interestingly, amyloid aggregates are often composed of proteolytic fragments, derived from the degradation of precursor proteins by yet unidentified proteases, which display higher amyloidogenic tendency compared to precursor proteins, thus representing an important mechanism in the onset of amyloid-based diseases. In the present review, we summarize the current knowledge on the proteolytic susceptibility of three of the main human amyloidogenic proteins, i.e., transthyretin, β-amyloid precursor protein, and α-synuclein, in the onset of amyloidosis. We also highlight the role that proteolytic enzymes can play in the crosstalk between intestinal inflammation and amyloid-based diseases.

## 1. Introduction

Amyloidoses are a group of rare diseases characterized by misfolding and extra- or intra-cellular accumulation in the form of highly organised fibrillary protein aggregates in a variety of tissues [1]. The fibers can build up in a single organ (i.e., localized amyloidosis) or in multiple organs (i.e., systemic amyloidosis), such as kidney, heart, or liver, and can also accumulate in the nervous system tissue. In localized amyloidoses, the deposit forms close to the precursor synthesis site; in systemic amyloidoses, the deposit may either form locally or at a distance from the precursor-producing cells. Amyloid aggregates and their preceding intermediates can cause proteotoxic intracellular stress and direct cell damage, leading to apoptosis, while amyloid fibril deposits disrupt tissue architecture, leading to the progressive failure of affected organs [2]. The global incidence of amyloidoses is estimated at five to nine cases per million patient-years, but given the complex nature of these diseases and the difficulties in diagnosis, there are no reliable large-population registries from which to derive accurate incidence and prevalence rates.

Within protein conformational diseases, amyloidoses represent the largest group of misfolding diseases, whereby specific peptides or proteins are converted from their soluble functional states into toxic and highly insoluble amyloid fibrils that can deposit in human tissues. Such aggregates arise from the failure of a specific peptide or protein to adopt its native conformation with a subsequent reduction in the amount of protein that is available to play a specific physiological role and increasingly high toxic effects [3]. These pathological states, although known for more than 150 years, have only recently been increasingly recognized in the pathogenesis of many human diseases, with dramatic social and medical burdens.

Traditionally, amyloidoses can classify into (i) primary amyloidosis, with no evidence of preceding or coexisting disease; (ii) secondary amyloidosis, correlated with chronic inflammatory or infectious conditions; (iii) familial hereditary amyloidosis, caused by inheriting gene mutations; and (iv) senile amyloidosis, which is related to aging. Amyloidoses are now referred to with a capital A letter (for Amyloid) followed by an abbreviation for the protein involved in fibril formation [4]. Table 1 reports the nomenclature and details the key features of the most common types of amyloidosis.

### 1.1. Clinical Features of Amyloid Diseases 

The clinical symptoms of amyloidosis depend on the organ(s) which are involved in amyloid fibril formation (Table 1). For instance, AL amyloidosis occasionally occurs with multiple myeloma, lymphoma, or Waldenstorm’s macroglobulinemia. Symptoms can occur in any organ of the body and include heart failure, protein in the urine, or kidney failure, enlarged liver, neuropathy or enlarged tongue. Likewise, AA amyloidosis usually begins in the kidneys, but other organs can subsequently be affected. In addition, symptoms of ATTR disease are usually neuropathy and cardiomyopathy and occur in mid to late life. We report the common clinical features of amyloidosis below.

Cardiac events are the most frequent cause of morbidity and mortality in amyloidosis by immunoglobulin light and heavy chain, apo-serum amyloid A, TTR and apo-lipopro-tein A I [5]. Patients with cardiac involvement have rapid and progressive congestive heart failure or arrhythmias. Heart amyloid depositions are characterized by restrictive cardiomyopathy, associated with disproportionate signs of right ventricular failure. Less frequently, in the setting of amyloid deposition in the small vessels of the heart, patients can have anginal symptoms but a normal coronary angiogram [6].

Kidneys are the organs most frequently affected by AL, AA, AFib, ALECT2, ALect2, AApoA, and AApoC amyloidosis, where glomeruli are primarily affected, with progressive nephrotic syndrome and renal failure [7]. The consequence is a dialysis-dependent life. In some cases, such as AL amyloidosis, albuminuria occurs with the alteration of normal diuresis. In addition, amyloid deposition in the tubulointerstitial can cause progressive renal damage [7].

Neuropathy, either peripheral or autonomic, is a failure caused by amyloid deposition in the nerves [8]. This evidence is a feature of AL amyloidosis and some hereditary types of ATTR and AApoA1 amyloidosis. Peripheral neuropathy is predominantly axonal and involves both small and large fibers. Patients with peripheral neuropathy present loss of sensation, the absence of deep tendon reflexes, and pain, while autonomic neuropathies are characterized by orthostatic hypotension, gastrointestinal inconvenience, early satiety, and impotence in men.

In AL, AH and AA amyloidosis, pulmonary and airway amyloidosis, which can occur in both systemic and localized diseases, are typically correlated with dyspnoea, haemoptysis, or hoarseness. Amyloid can deposit in four different areas of the lung, i.e., interstitial, nodular, pleural, and tracheobronchial [9]. Most commonly, submucosal deposition in the tracheobronchial airway results in airway obstruction, recurrent pneumonia, hoarseness, and segmental collapse [10]. Notably, diffuse interstitial disease presents with functional deterioration, although this decline is more commonly due to cardiac amyloidosis [9].

Soft tissues can be involved as well, and it is almost distinctive to AL amyloidosis. For instance, macroglossia, muscular pseudohypertrophy, the enlargement of salivary glands, and submandibular soft-tissue infiltration are a common manifestation of amyloidosis [11].

### 1.2. Amyloid Fibrils Composition

The fibrillar material which is found in amyloid deposits in vivo derives from several different and often unrelated precursor proteins, characterized by different size, amino acid sequence, three-dimensional structure, and function [12,13]. Furthermore, the recent use of high-resolution mass spectrometry approaches to identify amyloids suggests that many more proteins might be amyloidogenic [14,15].

Despite the different chemical and structural properties of the precursor proteins, amyloid deposits are composed of protein fibrils that share a remarkably similar structure, with a diameter of 7–13 nm, as observed by electron microscopy or atomic force microscopy techniques [12], and a common core structure consisting of anti-parallel β-strand (less commonly, parallel β-strands) that form extended sheets [16]. They generally comprise 2–8 protofilaments, each approximately 2–7 nm in diameter, that repeatedly twist around each other or associate laterally as flat ribbons 2–7 nm high and up to 30 nm wide [17]. However, mono-protofilament fibrils have also been observed [17,18]. The existence of such fibrillar ultrastructures allows the regular intercalation and binding of spectroscopically active dyes, such as thioflavin-T (ThT) and Congo red [19] representing useful diagnostic tools for assessing amyloid formation. Different features are widely accepted as hallmarks of amyloid structure, such as (i) fibrillar morphology detected by microscopy techniques, (ii) cross β-structure, (iii) characteristic dye binding properties, and (iv) proteinase-K resistance [20]. It has been shown that amyloid deposits in vivo also contain several minor non-fibrillar constituents, including serum amyloid P component (SAP) and glycosaminoglycans (GAGs) [7,21] that help to stabilize amyloid fibrils. SAP is a plasma glycoprotein synthesized in the liver, which is able to reversibly bind (K_d_ ~ 1 µM) to all types of amyloid fibrils with a calcium-dependent mechanism and protects fibrils towards proteolysis and phagocytosis both in vivo and in vitro [22,23]. GAGs are negatively charged polysaccharides that can strongly bind to fibers, promoting the initial steps of fibril nucleation by creating a scaffold for further fibril assembly [24]. 

It is widely accepted that the molecular mechanism undergoing the generation of amyloid fibrils starts from substantial conformational changes and the partial unfolding of native protein structures that can lead to the exposure of aggregation-prone regions. The process involves stepwise nucleation and growth phases [25,26]. At the first stage, protein monomers, completely or partially disordered, can convert into intermediate species, called nuclei, through a thermodynamically unfavorable process. Next, fibrils can grow and elongate from these nuclei through the addition of other monomeric molecules [12]. Hence, nuclei can be considered as the smallest structures able to initiate fibril elongation, or the smallest species in which the rate of further monomer addition exceeds that of monomer release to form oligomers [27].

Oligomers are prefibrillar species and play a key role in amyloidogenesis, as they represent the most pathogenic species in the diseases associated with amyloid fibril formation [28]. When aggregation is initiated in intrinsically disordered systems, the initial oligomers appear at first to adopt a disordered structure with more highly organized oligomers appearing later [15]. Such early aggregates are typically small, do not (or only weakly) bind amyloid-specific dyes, do not exhibit a significant content of β-sheet structure, and have a looser and less compact structure than native protein structures. As aggregation proceeds, oligomers undergo structural arrangement into species that become more compact. Only at later stages these species convert to highly regular, in-register parallel cross-β structures to assume the final fibrillar morphology [12].

## 2. Proteolysis-Driven Amyloidosis

Besides those factors which have been widely recognized to promote amyloid fibril formation, such as genetic mutations, environmental factors, excessive concentration of the precursor protein, chemical modifications [29,30,31,32] or post-translational modifications [33], in vivo fragmentation of the precursor proteins by endogenous proteases is one of the most prominent aspects that characterize amyloid fibrils. On one hand, proteolysis has been implicated in amyloidogenesis in distinct forms of amyloidosis [29,30]; on the other, post-deposition digestion may be implicated in the degradation of pathogenic aggregates [31]. The aberrant proteolysis of precursor proteins leads to the formation of large fragments which are generally partially unfolded, less stable than the parent proteins, and display a much higher tendency to form self-aggregating folding intermediates, which evolve toward oligomers and, subsequently, amyloid fibers. Under physiological conditions, these abnormal or misfolded proteins are degraded by the proteasome pathway intracellularly, and by macrophages extracellularly [32]. It is thought that in amyloidosis, these control mechanisms are less efficient, resulting in the pathologic unbalancing of fibril production and degradation. In the following, we report data from the literature and from our laboratory showing that proteolytic enzymes, both circulating in the bloodstream or resident in specific tissues, can play a relevant role in vivo in the onset of amyloid diseases. Due to space limitations, we limit our discussion to the impact of proteolysis in the onset of genetic and acquired amyloidosis by transthyretin (TTR), α-synuclein (αSyn), and β-amyloid precursor proteins (APP).

### 2.1. Proteolysis of Transthyretin

**Figure 1:**
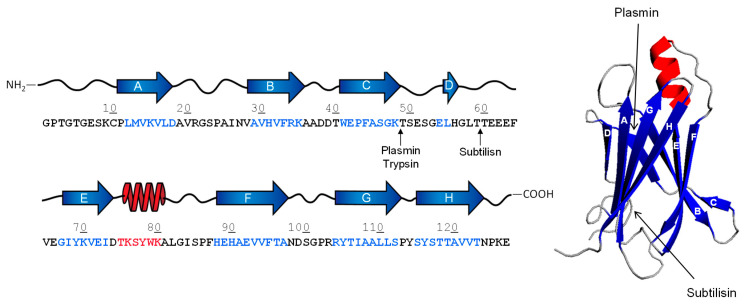
Schematic representation of proteolytic cleavage sites (↑) in TTR. The 3D structure (1tta.pdb) (**right panel**), the secondary structure and amino acid sequence (**left panel**) of monomeric TTR are shown. β-Strands and α-helices are indicated by blue arrows or red spirals, respectively.

TTR is a 55-kDa homo-tetrameric protein, synthesized in the liver and brain and abundantly present in human plasma [33]. TTR transports thyroid hormones in the blood and cerebrospinal fluid [33] that are bound to a central hydrophobic channel, positioned in the middle of the tetramer in the intersubunit space. Each TTR monomer consists of 127 amino acids and forms eight β-strands, named from A to H, which are arranged in a β-sandwich of two β-sheets (I and II) and one small α-helix. Each β-sheet is formed by four strands (β-sheet I: HGAD strands; β-sheet II: CBEF strands), while the α-helix is located between strands E and F [34,35,36] (Figure 1). The monomers tightly interact with each other to form stable dimers, mainly stabilised by extensive hydrogen bonds. The two dimers weakly interact to form the final tetrameric structure, which is predominantly stabilised through hydrophobic interactions involving the A-B and G-H loops [34,35,36].

TTR aggregation causes amyloidosis, which is associated with two different pathological conditions, i.e., hereditary familial amyloidosis (fATTR) and acquired senile systemic amyloidosis (sATTR). fATTR is a rare disorder with an early age of onset, characterized by the deposition of TTR amyloid fibrils in different organs, leading to polyneuropathy and/or cardiomyopathy [37,38]. It has been recognized as the most common cause of hereditary amyloidosis worldwide [39]. fATTR is caused by single point mutations affecting the TTR gene. More than 100 amyloidogenic TTR mutations are known, while the most prevalent are Val30Met (common in Portugal, Japan, and Sweden), Val122Ile (carried by African Americans) and Ser52Pro. The mutant Val30Met has high penetrance, early age of onset (twenties to early forties), and rapid progression of polyneuropathy [40]. The Val122Ile mutation is associated with a higher frequency of congestive heart failure and a trend towards lower mortality [41]. Individuals carrying the Ser52Pro mutation develop aggressive and early-onset fatal ATTR. Conversely, sATTR involves wild-type TTR, more often affects elderly people, and is usually associated with cardiomyopathic complications. It has been recognized as an “underappreciated” cause of heart failure in 1–3% older adults >75 year of age [42].

TTR aggregation occurs after dissociation of the tetramer into monomer subunits, which, upon partial unfolding, undergo self-association into amyloid aggregates [43]. Interestingly, experimental evidence suggests the existence of different types of TTR amyloid fibrils in a variety of tissues. Amyloid deposits may be composed of a mixture of cleaved and full-length TTR, identified as type-A and type-B fibrils. Typically, type-A fibrils are rich in TTR fragments that span the C-terminal region 46–127, with fragment 49–127 being the most abundant, whereas intact TTR is only scarcely represented. Conversely, full-length TTR is highly represented in type B fibrils [39,44,45]. Different amyloidogenic fragments may be found in various tissues. For example, the vitreous TTR appeared to be fragmented between Lys48-Thr49 residue, while cardiac TTR may be cleaved at multiple sites in the peptide segment encompassing the amino acid sequence 46–52 [46] (Figure 1). Several other shorter C-terminal fragments have been identified in TTR amyloid deposits [47]. However, the C-terminal fragment 49–127 is the main component of ex vivo TTR amyloid fibrils in tissue biopsies of cardiac deposits [44,48]. It has been reported that fibrils formed by proteolytic fragments are most often found in old patients with sATTR, suggesting that age-associated alteration of TTR proteolysis is a risk factor for amyloidosis.

The protease responsible for TTR cleavage has not yet been safely identified. However, the high specificity fragmentation pattern suggests that it could be a trypsin-like protease. Some evidence indicates that digestive (i.e., trypsin) [39] and fibrinolytic (i.e., plasmin) [49] proteases of the chymotrypsin family are able to cleave TTR under shear stress conditions in vitro, and generate the amyloidogenic fragment TTR (49–127), which is abundantly present in both sATTR and fATTR patients’ amyloid deposits [39,50,51]. Recent findings from our laboratory indicate that subtilisin, a serine protease secreted from *Bacillus subtilis* (a non-pathogenic component of the normal gut microbiota) [52], can efficiently cleave TTR generating the amyloidogenic fragment TTR (59–127) in vitro [53], which is resistant to further proteolysis and forms typical amyloid fibrils, as detected by ThT binding and transmission electron microscopy analysis [53]. In human plasma, subtilisin can efficiently cleave TTR, escaping the inactivation of protease inhibitors in vivo. Importantly, the same fragment TTR (59–127), derived from both wild-type and Val30Met mutant, was identified in vivo in the amyloid deposits of patients with fATTR [47,51]. It is noteworthy that TTR C-terminal fragments, i.e., TTR (49–127) and TTR (59–127), are more prone to form amyloids in vitro than the full-length protein. Indeed, natural or recombinant wild-type TTR form fibrils after prolonged incubation under mild denaturing conditions (i.e., 37 °C, pH 4.4, high shear stress conditions), with a lag phase of ~5 h, whereas fibril formation by TTR C-terminal fragments is much faster (lag phase: 15 min) and, more importantly, occurs under physiological (non-denaturing) conditions (i.e., 37 °C, pH 7.4, static conditions) [53]. The structural basis for the higher amyloidogenic potential of TTR fragments can reside in the partially unfolded structure they assume in solution, compared to the compact folded structure of TTR native tetramer, as documented by circular dichroism and hydrogen-deuterium exchange mass spectrometry measurements (HDX-MS) [53].

Intriguingly, the increased permeability of the intestinal mucosa, often observed in aged people [54,55], allows subtilisin (and likely other bacterial proteases) to pass across the gut mucosa into the bloodstream and cleave TTR to generate amyloidogenic fragments, which can deposit infiltrative fibrils in the heart of sATTR patients. In fact, patients with TTR amyloidosis present higher plasma proteolytic activity [2,56]. The same condition was also observed in patients characterized by the moderate chronic inflammatory state and intestinal microbiota dysbiosis [55]. Likewise, the pathological increase of intestinal permeability results in abnormally high blood trypsin concentrations and presumably the generation of the amyloidogenic fragment TTR (49–127), which is ubiquitous in TTR amyloid deposits. Trypsin, in fact, is not exclusively localized at the level of the duodenum and can undergo intestinal re-absorption from the small intestine to the bloodstream [57,58]. Recent studies showed that amorphous protein aggregates can stimulate plasminogen activation to plasmin, which can proteolytically degrade the aggregates and release smaller soluble protein fragments, which in turn are cytotoxic in vitro for both endothelial and microglial cells [59]. The presence of plasmin in the bloodstream, its structural similarities to trypsin, and the reported activation of plasminogen activation system in other amyloid-related disorders, such as Alzheimer’s disease (AD) [60] and immunoglobulin light chain amyloidosis [61,62,63], suggest that plasmin could play a substantial key role in TTR amyloidogenesis. It has been proposed that circulating TTR can diffuse to the extracellular compartment and be entrapped in the fibrin clot [49]. Upon plasminogen activation, TTR may be variably cleaved to generate a mixture of both truncated and full-length TTR, which ultimately could assemble into amyloid fibrils and deposit in the extracellular space [49]. Although it has yet to be firmly established whether TTR fragmentation occurs prior to or after aggregation and where it occurs, i.e., in the circulation or at the site of deposition, an increase of proteolytic activity in the plasma from fATTR patients has been measured in comparison to healthy controls, suggesting that the proteolytic process could happen in the bloodstream before fibril formation [56].

### 2.2. Proteolysis of β-Amyloid Precursor Protein

**Figure 2:**
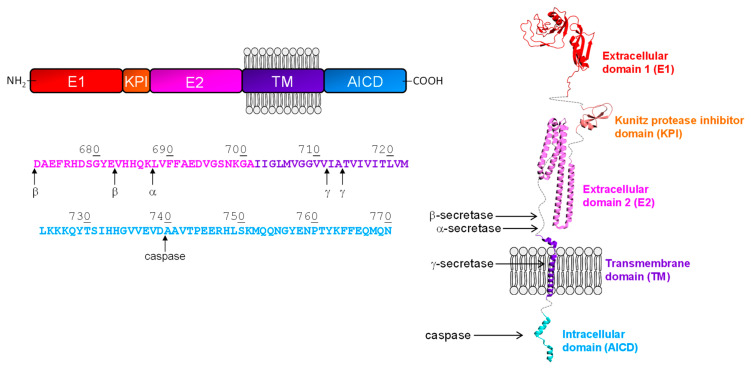
Schematic representation of domain organization and proteolytic cleavage sites (↑) in APP (**left panel**). The domain architecture and amino acid sequence of the APP are highlighted. E1 and E2 indentify the extracelllar domains, KPI is the Kunitz-type domain, while TM is the trans-membrane domain and AICD is the APP intracellular domain. Schematic representation of the APP threedimensional structure (**right panel**). The protein cartoon is obtained by combining different crystal structures (1zjd.pbd; 2llm.pdb; 3dx0.pdb; 3kzm.pdb; 3umk.pdb) of APP domains. The arrows approximately indicate where the cleavage sites are found on the APP structure.

Beta amyloid precursor protein (APP) is a type I integral membrane protein that is expressed in many tissues, especially in the synapses of neurons, where it plays a central role in the pathogenesis of AD. APP consists of a large extracellular glycosylated N-terminus, a hydrophobic transmembrane domain, and a shorter cytoplasmatic C-terminus (Figure 2). Although the physiological role of APP has yet to be firmly established, earlier studies show that it acts as a regulator of synaptic formation and repair [64], and plays a key role in anterograde neuronal transport [65] and iron export [66]. Alternative splicing of the APP transcript generates eight isoforms, of which three are the most common, i.e., the 695 amino acid form, which is expressed predominantly in the central nervous system (CNS), and the 751 and 770 amino acidic forms, which are more ubiquitously expressed, in peripheral cells and platelets [67]. Notably, APP695 differs from other longer forms by lacking the Kunitz-type protease inhibitor sequence in its ectodomain [68].

APP undergoes proteolytic processing, which is fundamental for generating amyloidogenic species (i.e., Aβ peptides) that can locally accumulate at the level of extracellular plaques in the brain. The newly-generated fragments can aggregate into various types of assemblies, including oligomers and amyloid fibrils. Oligomers are soluble species composed of eight molecular species (100–200 kDa), characterized by an extended coil or β-sheet structure [69], and can spread through the brain. Amyloid fibrils are larger and insoluble and can also assemble into plaques, forming histological lesions characteristic of AD. APP proteolytic events involve several different soluble and membrane-bound proteases (denoted as secretases) via two major pathways (Figure 2). In the non-amyloidogenic pathway, APP is sequentially cleaved by α-secretase and γ-secretase. α-Secretase cleavage involves the residues Lys688-Leu689 in the Aβ peptide sequence, releasing the large secreted extracellular domain (sAPP-β) and the membrane-bound 83-amino acid C-terminal APP fragment (C83). APP C83 is further processed by -secretase to release the P3 peptide and the APP intracellular domain (AICD), both of which are rapidly degraded [70]. The amyloidogenic processing of APP involves sequential cleavage by β- and γ-secretase at N- and C- termini of Aβ peptide, respectively [71]. β-secretase cleaves at two residues (Asp672 or Glu682) at the N-terminal of the Aβ peptide sequence, shedding sAPP-β and generating a membrane-associated C-terminal fragment composed of 99 amino acids (C99). Subsequently, γ-secretase processes C99 at multiple sites to produce cleavage fragments, from 43 to 51 amino acids long, that are further cleaved to the main final Aβ forms, i.e., the Aβ40 and the Aβ42, which at concentrations >50 nM aggregate and fibrillate to form amyloid plaques in the brain [72,73]. In addition, γ-secretase also releases AICD, which can translocate into the nucleus where it can act as a regulator of gene expression, apoptotic events, and cellular calcium homeostasis. Finally, AICD is degraded by caspases to produce the neurotoxic peptide (C31) [74].

APP β-secretase (β-site APP Cleaving Enzyme, BACE1) is highly expressed in AD patients, where it may accelerate the amyloidogenic pathway in the brain and impair neuronal survival [75]. BACE1 is a transmembrane aspartic protease, 500 residues in length, with two active sites located on the luminal side of the membrane. This allows the enzyme to readily access to its substrate within the lumen of the Golgi vesicles, where it competes against α-secretase for APP cleavage, or within endosomes and lysosomes [76]. γ-Secretase is a transmembrane complex consisting of at least four proteins: presenilin, nicastrin, anterior pharynx defective-1 and presenilin enhancer-2. During the maturation of the complex, presenilin is endoproteolytically cleaved to form an N-terminal and a C-terminal fragment that both contain aspartyl protease sites that tightly are required for the activity of the mature enzyme. The other components may modulate enzyme activity in response to physiological stimuli. The components of the γ-secretase complex are widely expressed in a variety of tissues of the brain including liver, heart, and lung [77,78]. All presenilin-associated proteins are embedded in the membrane [79]. The substrate passes in between the two presenilin fragments, which appear to form a hydrophilic pocket in the membrane that allows the proteolytic cleavage [80,81]. Presenilin mutations appear to be the main cause of familial AD [82], suggesting a key role for the γ-secretase complex in disease onset.

Aβ peptides are intrinsically unstructured, but the C-terminal portion seems to assume an α-helical organization that results it being particularly metastable undergoing a global conformational rearrangement that allows the formation of β-sheet structure [83]. For this reason, the C-terminal region is thought to drive protein aggregation in AD.

### 2.3. Proteolysis of α-Synuclein

α-synuclein (αSyn) is a small (14 kDa) acidic protein that is highly conserved in vertebrates and implicated in the pathogenesis of Parkinson’s disease (PD) [84]. It is abundantly present in vivo in the CNS and in the nuclei of neuronal cells and presynaptic terminals, where it binds to synaptic vesicles and modulates vesicle homeostasis and synaptic plasticity [85]. Specific mutations (i.e., Ala30Pro, Glu46Lys, and Ala53Thr) and the multiplication of the wild-type gene were indeed found in some early-onset familial PD patients [84]. αSyn is a structurally disordered monomeric protein, either when isolated in solution [86] or in cellular environments, where it assumes a loosely packed, dynamic structure [87].

**Figure 3:**
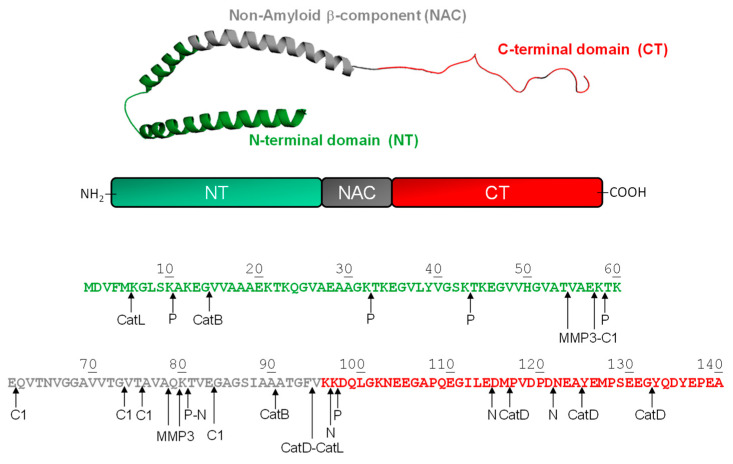
(**Top panel**) Schematic representation of membrane-bound structure (1xq8.pdb) of αSyn; NT is the N-terminal domain 1-60, NAC refers to the Non-Amyloid Component 61-95, and CT corresponds to the C-terminal region 96-140. (**Bottom panel**) Domain architecture, amino acid sequence and proteolytic cleavage sites (↑) of αSyn by different proteases, i.e., calpain-1 (C), cathepsin (Cat) B, D and L, matrix metallo-proteinase-3 (MMP3), neurosin (N), and plasmin (P).

Although αSyn is considered a cytoplasmatic protein, it has been detected in extracellular biological fluids (in monomeric and oligomeric forms), including human cerebrospinal fluid and the blood plasma of healthy and sick individuals [88,89,90]. Structurally, αSyn consists of three distinctive regions (Figure 3), i.e., the N-terminal (1–60), the Non-Amyloid β-Component, NAC, (61–95), and the C-terminal region (96–140).

The N-terminal domain is positively charged and assumes a helical conformation on the lipid membranes [91]. More than half of the N-terminal region is characterized by seven imperfect repeats of eleven amino acids, each having a conserved six-amino-acid core with the consensus sequence KTKEGV that represents the key mediators of normal protein tetramerization [92]. NAC is highly hydrophobic, has strong β-sheet conformational propensity, and mediates αSyn aggregation/fibrillation [93]. The C-terminal region is strongly negative and has been proposed to inhibit αSyn aggregation/fibrillation by electrostatic repulsion [94]. NAC from αSyn is the second major component of amyloid plaques in AD [95]. In fact, this region exhibits a significant degree of similarity with the Aβ peptide, which also contains a highly hydrophobic domain. In vitro studies have shown that NAC presents a β-sheet secondary structure and forms ordered fibrils very similar to those formed Aβ [96,97]. Upon prolonged incubation, αSyn aggregates and forms amyloid fibrils [98], characterized by a cross-β-sheet structure and stabilized by an extensive hydrogen bond network [99]. αSyn amyloid aggregates can then accumulate in the dopaminergic neurons of the brain’s *substantia nigra* and are considered as a key neuropathological hallmark of PD [84,100,101].

αSyn undergoes various post-translational modifications, such as phosphorylation, ubiquitination, nitration, glycation, SUMOylation, and proteolytic degradation. In fact, αSyn fragments have been detected in the brain of healthy individuals and in the Lewy bodies of PD patients, where they act as seeds for aggregation and become neurotoxic due to the high propensity to aggregate [102]. Two proteolytic processes are involved in αSyn degradation: the cytosolic ubiquitin/proteasome pathway and the autophagic/lysosomal pathway, including macro-autophagy and chaperone-mediated autophagy [103,104]. As a result, different proteases (i.e., extracellular, cytosolic and lysosomal) can participate in αSyn proteolytic degradation.

Among the extracellular proteases, plasmin, neurosin, and matrix metalloproteinase-3 (MMP-3) play a key role in αSyn processing. Plasmin cleaves monomeric and aggregated forms of αSyn (oligomers and fibrils) in a dose- and time-dependent manner [105], mainly at the N-terminal region, generating six major C-terminal fragments, from 11–140 to 98–140 (Figure 3). All plasmin cleavage sites are located after a lysine residue (i.e., Lys10, Lys32, Lys43, Lys58, Lys80, and Lys97) and are mostly within the KTKEGV repeat region. The resulting truncated species are unable to activate neighboring astrocytes and/or microglia [105], suggesting that plasmin may have a beneficial effect on the pathogenesis of PD. Neurosin, another trypsin-like serine protease highly expressed in the brain of PD patients [106], cleaves αSyn in the NAC region, at Lys80-Thr81 bond, and in the C-terminal region, at the peptide bonds Lys97-Asp98, Glu114-Asp115, and Asp121-Asn122. The resulting fragments prevent the aggregation of αSyn by reducing the concentration of the intact protein and by inhibiting protein polymerization [106]. MMP-3, a stromelysisn endopetidases synthesized by neurons, microglia, and astrocytes, can generate four major N-terminal αSyn fragments, i.e., 1–54, 1–57, 1–78 and 1–79 and their C-terminal counterparts, as well as some minor fragments resulting from cleavage at the Ala91-Ala92 and Gly93-Phe94 bonds [107] (see Figure 3). The major cleavage sites occur in the NAC domain (i.e., Thr54-Val55, Glu57-Lys58, Ala78-Gln79, and Gln79-Lys80). Interestingly, MMP-3 usually generates fragments that retain the ability to form aggregates which are characterized by shorter fibrils and more spherical granules [108].

Calapin-1, a cytosolic calcium-activated neutral protease, generates four main αSyn N-terminal fragments, i.e., 1–57, 1–61, 1–73, 1–75, and 1–83 [109,110]. The corresponding cleavage sites cluster with or close to the NAC domain, resulting in the generation of species with reduced aggregation propensity. Intriguingly, these fragments are generated from soluble αSyn, but not from fibrillar αSyn. Fibers of αSyn, and especially the mutant Ala53Thr, are predominantly cleaved within the C-terminus at residues Glu114 and Asn122, indicating a different contribution of calpain-1 to αSyn proteolysis under physiological and pathological conditions [109].

Cathepsin-D, a lysosomal aspartyl protease implicated in the pathogenesis of various neurovegetative disorders, can degrade αSyn, generating an amyloidogenic C-terminally truncated fragment, αSyn(1–94) [111]. The primary cleavage site Phe94-Val95 is followed by other minor cuts at Met116-Phe117, Ala124-Tyr125, and the Gly132-Tyr133 bonds. Notably, cathepsin-D requires the presence of anionic phospholipids to degrade αSyn. Recently, cathepsin-B and cathepsin-L have been identified as new enzymes involved in αSyn lysosomal degradation [112]. Cathepsin-B and cathepsin-L are cysteine protases, associated with different neurodegenerative amyloid diseases [113], and are identified in liver lysosomal extracts [112]. Cysteine cathepsins are capable of proteolyzing the central and amyloidogenic region of αSyn, at the peptide bonds Gly14-Val15 and Ala90-Ala91, and Met5-Lys6 and Phe94-Val95, for cathepsin-B and cathepsin-L, respectively. In contrast to cathepsin-D, soluble, membrane bound and aggregated αSyn are readily digested by both cathepsin-B and cathepsin-L. Whereas cathepsin-D activity is stimulated by anionic lipids in vitro, cathepsin-L is still the most efficient, with cathepsin-D comparable to cathepsin-B activity under these conditions. Overall, these results suggest that cysteine cathepsins are essential in the lysosomal degradation of αSyn.

## 3. Generation and Amyloid-Forming Properties of Proteolytic Fragments

**Figure 4:**
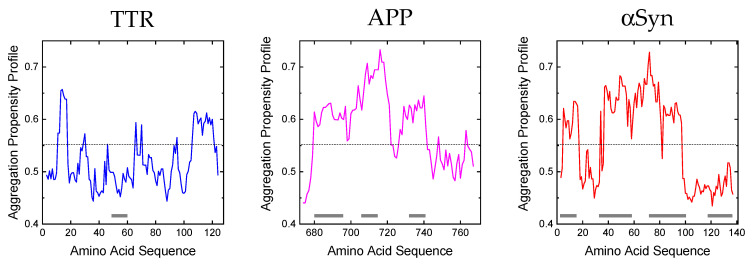
Amyloid aggregation profiles of TTR, APP and αSyn. The predicted aggregation propensities were obtained using the MetAmyl tool [121]. Regions of low structural stability correspond to the highest peaks in the aggregation propensity profiles (hexapeptide amyloidogenicity). The grey bars represent the regions susceptible to proteolytic attack, to generate amyloidogenic species.

Experimental evidence suggests that several characteristics of the polypeptide chain are important for amyloid formation, including length, charge [114,115], hydrophobicity [116,117,118], patterns of polar and nonpolar residues [119], and propensity to adopt diverse secondary structures [116]. In fact, polypeptide sequences that can originate fibrils appear to contain local ‘sensitive’ regions that are particularly susceptible to proteolytic degradation and aggregation [116], and that are part of the β-sheet core in the resulting protofibrils and fibrils. Furthermore, other protein regions are known for their propensity to induce aggregation after single-point mutations [12], which can alter their physico-chemical properties. The resulting mutants become pathogenic primarily due to an enhanced rate of aggregation and/or for their higher susceptibility to proteolytic cleavage (e.g., the αSyn mutant Glu46Lys). Notably, at physiological pH, residues such as Trp, Phe, Cys, Tyr, and Ile in a polypeptide chain have the highest amyloid propensity, while Asp, Lys, Glu, and Arg have the lowest [120]. Whereas these properties are valid for unfolded polypeptide chains (i.e., Aβ peptides and αSyn), in the case of globular proteins (i.e., TTR) the propensity to form amyloid structures is in general inversely related to the stability of their native states. However, many of the proteins associated with amyloidosis are at least partially unfolded under physiological conditions or become only loosely structured after proteolysis. Due to the complexity of the system, different computational algorithms have been designed to predict with reasonable accuracy the amyloid-forming propensity of proteins/fragments [120]. Figure 4 shows the aggregation propensity profiles for the tree proteins considered in this review as generated by the MetAmyl [121] predictive algorithm. Interestingly, from this analysis, it appears that proteolytic nicking occurs at protein segments characterized by higher conformational flexibility and/or amyloidogenic propensity.

*Transthyretin*. The aggregation profile of TTR is not characterized by significant regions with high amyloidogenic tendency, but only by three segments with a lower propensity, i.e., residues 10–17, 65–70 and 106–121. Due to its globular/compact nature, TTR must be destabilized in order to possibly aggregate [122,123]. Our hydrogen/deuterium exchange (HDX) mass spectrometry data [53] indicate that, in addition to the N-terminal and C-terminal regions, TTR contains a segment (residues 43–61) with high flexibility and high intrinsic disorder probability that can be attacked by proteolytic enzymes (i.e., plasmin, trypsin or subtilisin) to generate amyloidogenic fragments. In the case of subtilisin, for example, the first cleavage at the Leu58-Thr59 peptide bond destabilizes the newly formed N-terminal fragment (1–58), which can be further attacked by the protease. On the contrary, the resulting C-terminal fragment (59–127) is more stable to further proteolysis by subtilisin and starts to aggregate and generate amyloidogenic fibers. In fact, HDX and nuclear magnetic resonance spectroscopy (NMR) [124] on amyloid recombinant TTR fibers indicate that only two (C loop residues 41–49 and D loop residues 53–55) of the eight native β-strands are exposed to the solvent and are not part of the fibrillar core and therefore can more likely be proteolyzed.

*β-amyloid precursor protein*. The aggregation profile of APP reveals two regions of high amyloidogenicity propensity, i.e., the central region (residue 680–720) and the C-terminal region (residue 726–740). Both regions are susceptible to proteolytic attack (by β-, α-, γ-secretase, and caspase) and play an important structural role in the aggregation tendency. Mutagenesis experiments indicate that the sequence 686–692 (15–21 in Aβ42 peptide numbering) is particularly important in the process of amyloid formation [125]. This is in fact part of the structural core of fibrils, as detected by NMR and electron paramagnetic resonance (EPR) coupled to site-directed spin labelling [126,127], and they have a well-defined β-sheet structure. The region immediately before the C terminus, spanning the residues 701–708 (31–37 in Aβ42 peptide numbering), is also important for aggregation [125]. Additionally, the peptide corresponding to residues 705–712 (34–42 in Aβ42 peptide numbering) can readily form an ordered β-structure [128] and becomes part of the core in the fibrillar aggregate.

*α-synuclein*. The aggregation propensity calculated for αSyn shows that the regions with the highest propensity for amyloidogenesis are 36–40, 45–53, and 61–95. In particular, most of the cleavage sites are localized in the correspondence of the NAC region (61–95), which results in the most prominent segment for the generation of fragmented amyloidogenic species. In fact, NMR [129], limited proteolysis [130], and EPR [131] data indicate that this central region forms the core of αSyn fibrils. Recent pulsed HDX data [132] confirm that the aggregation involves the core region of αSyn, while the N-terminal contribution to the aggregation is moderate and the C-terminus remains solvent-accessible throughout the process. Therefore, in this case, the proteolytic attack occurs in correspondence of the amyloidogenic sequence.

## 4. Conclusions and Perspectives

Proteolysis is one of the common post-translational modifications and highly preferentially occurs both in vitro and in vivo at the most accessible and flexible sites (i.e., turns and loops) in proteins, whereas rigid secondary structure elements (i.e., a-helices and b-sheets) are usually resistant to proteolytic attack [133]. These remarkable differences in susceptibility to proteolysis in different protein regions arise from the fact that, in order to be cleaved, peptide segments must “adapt” to the substrate recognition sites on the protease structure, and the “energetic cost” for this adaptation is lower in conformationally flexible protein regions and much larger in secondary structure elements, which should first locally unfold, after internal hydrogen-bonds breakage, and then properly bind to the substrate recognition sites of the protease [133]. The analysis of data form our laboratory and from the literature on relevant protein systems (i.e., TTR, APP and αSyn) indicate that the proteolysis of precursor proteins is instrumental for the generation of proteolytic fragments with higher amyloid-forming tendencies that have been found in amyloid deposits in vivo. Therefore, besides their intrinsic propensity to form amyloid fibrils, the proteolysis-driven aggregation of proteins can be considered as an interesting mechanism in the pathogenesis of amyloid diseases.

In this review, we have focused on the generation and amyloid proteins of proteolytic peptides that originated from TTR, APP and αSyn. In the case of TTR, protreolytic cleavages promoted by plasmin, trypsin and subtilisin generate amylodogenic fragments which are known as the principal components of fibril deposits in sATTR and fATTR. APP-**cleaving** secretases promote the formation of Aβ peptides, which are the most aggregation-prone and are also the most abundant species in the plaques of AD patients. In the case of αSyn, the proteolytic cleavage at the NAC region (i.e., by neurosin and calapin-1) suppresses protein aggregation and exerts some beneficial effect on PD pathogenesis, while C-terminal degradation (i.e., by cathepsin) generates fragments with higher amyloidogenic potential.

Although for APP and αSyn the endogenous proteases capable to modulate the amyloidogenic potential of precursor proteins have been identified, in the case of TTR, the safe identification of the physiological proteases generating more potent amyloidogenic fragments is still awaited [39,49,53,134]. Furthermore, the increased permeability of intestinal mucosa, which has been found in elderly people and in patients with inflammatory bowel diseases, might allow for the translocation of proteases secreted from intestinal bacteria from the gut lumen to the bloodstream, where they can promote the cleavage of precursor plasma proteins and the generation of amyloidogenic fragments [59,60].

In this scenario, protease inhibitors that block the activity of selected (i.e., pro-amyloidogenic) proteases may represent a therapeutic option for reducing the production of amyloid-forming fragments. BACE1 inhibitors, for example, have recently reached clinical trials [135,136]. Another promising strategy to reduce protein fragment production or accumulation is based on oligonucleotide and RNAi therapies. Recently, both the European Medicines Agency and the Food and Drug Administration have approved an antisense oligonucleotide [137] and an siRNA [138,139] for the treatment of ATTR. In addition, affibodies (i.e., antibodies mimetics) have been shown to effectively sequester amyloidogenic peptides by encapsulating protein fragments in a tunnel-like cavity, thus preventing the formation of toxic aggregates [140]. 

Given the increasing incidence of amyloidosis in the world-wide population, protease inhibitors or bio-molecules which are able to interfere with precursor protein fragments’ generation and aggregation might be a promising therapeutic strategy.

## Figures and Tables

**Table 1:** Common types of human amyloidosis, as adapted from the International Society of Amyloidosis (ISA) official nomenclature [4].

Amyloidoses	Precursor Fibril Protein	Type of Amyloidosis		
		Systemic and/or Localized	Acquired and/or Hereditary	Organ or Tissue Affected
AL	Immunoglobulin light chain	S, L	A, H	all, no CNS ^a^
AH	Immunoglobulin heavy chain	S, L	A	all, no CNS ^a^
AA	Apo Serum Amyloid A	S	A	all, no CNS ^a^
ATTR	Transthyretin, wild type Transthyretin, mutants	S S	A H	heart, lung, ligaments, PNS ^b^, ANS ^c^, eye
Aβ2M	β2-microglobulin, wild type β2-microglobulin, mutants	S S	A H	musculoskeletal system, ANS ^c^
AApoAI	Apolipoprotein A I, mutants	S	H	heart, liver, kidney, PNS ^b^, testis, larynx, skin
AApoAII	Apolipoprotein A II, mutants	S	H	kidney
AApoAIV	Apolipoprotein A IV, wild type	S	A	kidney
AApoCII	Apolipoprotein C II, mutants	S	H	kidney
AApoCIII	Apolipoprotein C III, mutants	S	H	kidney
AGel	Gelsolin, mutants	S	H	kidney, PNS ^b^, cornea
ALys	Lysozyme, mutants	S	H	kidney
ALECT2	Leukocyte chemotactic factor-2	S	A	kidney
AFib	Fibrinogen α, mutants	S	H	kidney
ACys	Cystatin C, mutants	S	H	CNS ^a^, PNS ^b^, skin
ABri	ABriPP ^d^, mutants	S	H	CNS ^a^
Adan	ADanPP ^e^, mutants	L	H	CNS ^a^
Aβ	Aβ protein precursor, wild type Aβ protein precursor, variant	L L	A H	CNS ^a^
AαSyn	α-Synuclein	L	A	CNS ^a^
ATau	Tau protein	L	A	CNS ^a^
APrP	Prion protein, wild type Prion protein, mutants	L L, S	A H	CNS ^a^, PNS ^b^
ACal	(Pro)calcitonin	L, S	A	kidney, thyroid
AIAPP	Islet amyloid polypeptide	L	A	Langerhans’ islets, insulinomas
AANF	Atrial natriuretic factor	L	A	heart
APro	Prolactin	L	A	pituitary gland
AIns	Insulin	L	A	skin, muscle
ASPC	Lung surfactant protein	L	A	lung
ACor	Corneodesmosin	L	A	cornified epithelia, hair follicles
AMed	Lactadherin	L	A	senile aorta
Aker	Kerato-epithelin	L	A	cornea
ALac	Lactoferrin	L	A	cornea
AOAAP	Odontogenic ameloblast-associated protein	L	A	tooth forming tissues
ASem1	Semenogelin 1	L	A	*vesicula seminalis*
AEnf	Enfurvitide	L	A	skin
ACatK	Cathepsin K	L	A	kidney, angiomyolipoma
AEFEMP1	EGF-containing fibulin-like extracellular matrix protein 1 (EFEMP1)	L	A	portal veins

^a^ CNS: Central Nervous System; ^b^ PNS: Peripheral Nervous System; ^c^ ANS: Autonomic Nervous System. ^d^ ABriPP: amyloid Bri precursor protein; ^e^ ADanPP: Activity-dependent neuroprotective protein.

## Data Availability

Not applicable: in this study no new data were created.
